# Diagnosis and Treatment of Tremor in Parkinson’s Disease Using Mechanical Devices

**DOI:** 10.3390/life13010078

**Published:** 2022-12-27

**Authors:** Joji Fujikawa, Ryoma Morigaki, Nobuaki Yamamoto, Hiroshi Nakanishi, Teruo Oda, Yuishin Izumi, Yasushi Takagi

**Affiliations:** 1 Department of Advanced Brain Research, Institute of Biomedical Sciences, Graduate School of Medicine, Tokushima University, 3-18-15 Kuramoto-Cho, Tokushima-Shi 770-8503, Tokushima, Japan; 2 Department of Neurosurgery, Institute of Biomedical Sciences, Graduate School of Medicine, Tokushima University, 3-18-15 Kuramoto-Cho, Tokushima-Shi 770-8503, Tokushima, Japan; 3 Parkinson’s Disease and Dystonia Research Center, Tokushima University Hospital, 2-50-1 Kuramoto-Cho, Tokushima-Shi 770-8503, Tokushima, Japan; 4 Department of Neurology, Institute of Biomedical Sciences, Graduate School of Medicine, Tokushima University, 3-18-15 Kuramoto-Cho, Tokushima-Shi 770-8503, Tokushima, Japan; 5 Beauty Life Corporation, 2 Kiba-Cho, Minato-Ku, Nagoya 455-0021, Aichi, Japan

**Keywords:** Parkinson’s disease, essential tremor, diagnosis, stimulation, medical devices, machine learning

## Abstract

Background: Parkinsonian tremors are sometimes confused with essential tremors or other conditions. Recently, researchers conducted several studies on tremor evaluation using wearable sensors and devices, which may support accurate diagnosis. Mechanical devices are also commonly used to treat tremors and have been actively researched and developed. Here, we aimed to review recent progress and the efficacy of the devices related to Parkinsonian tremors. Methods: The PubMed and Scopus databases were searched for articles. We searched for “Parkinson disease” and “tremor” and “device”. Results: Eighty-six articles were selected by our systematic approach. Many studies demonstrated that the diagnosis and evaluation of tremors in patients with PD can be done accurately by machine learning algorithms. Mechanical devices for tremor suppression include deep brain stimulation (DBS), electrical muscle stimulation, and orthosis. In recent years, adaptive DBS and optimization of stimulation parameters have been studied to further improve treatment efficacy. Conclusions: Due to developments using state-of-the-art techniques, effectiveness in diagnosing and evaluating tremor and suppressing it using these devices is satisfactorily high in many studies. However, other than DBS, no devices are in practical use. To acquire high-level evidence, large-scale studies and randomized controlled trials are needed for these devices.

## 1. Introduction

Parkinson’s disease (PD) is a progressive degenerative disorder primarily characterized by the degeneration of dopamine neurons in the substantia nigra [1,2]. Its main symptoms include tremor, rigidity, bradykinesia, akinesia, and postural instability [3]. Tremors are one of the most common motor symptoms of PD. PD can be classified into different subtypes as follows: patients with predominant akinesia/rigidity, which is an akinetic-rigid type (ART), and those with a tremor-dominant type (TDT) [4]. PD-ART displays greater cognitive impairment and faster progression than TDT-PD [5]. This warrants understanding the status of tremors, considering their role in diagnosing the disease and its symptoms. Non-pathological, slight physiological tremors can be found in normal individuals. Pathological tremor affects more than 0.4% of the population [6], and its incidence and prevalence increase substantially with age [7]. 

Tremor is caused by a variety of conditions [8], and its exact underlying mechanism is not understood [9]. Among several causes of tremor, the most common and incidental types of tremor are seen in patients with PD and essential tremor (ET) [6]. ET is a major differential diagnosis. According to the 2018 Movement Disorders Consensus Criteria, ET is characterized by isolated bilateral upper limb movement tremor with a duration of at least 3 years without other neurologic signs [9]. Tremor in patients with ET and PD is sometimes confusing. PD is a complex neurodegenerative disorder, usually characterized by asymmetrical onset bradykinesia, muscular rigidity, postural instability, and tremor. Patients with PD present with resting tremor, as well as other symptoms, except during the early stages. Resting tremors are often enhanced by walking and performing tasks, such as calculation. In contrast, tremor severity tends to increase during kinetic tasks in patients with ET. Despite the lack of a test to confirm diagnosis, medical interviews, physical examinations, and blood tests should exclude other common causes of action tremors, such as the side effects of certain medications or hyperthyroidism.

Tremor assessment is based on physical examination by a neurologist. Current diagnostic methods and quantification are based on the phenomenological demonstration of tremor, principally with the help of movement disorder scales, such as the essential tremor rating assessment scale [10], Fahn–Tolosa–Marin scale [11], and Unified Parkinson’s Disease Rating Scale (UPDRS) [12]. The correct diagnosis of the different tremor types is essential for treatment, which may depend on the specific etiology of each type. However, tremor misdiagnosis owing to confusion between PD and ET can occur in 20% to 30% of cases [13,14]. Thus, technological solutions may improve the quality of diagnosis and quantify the disease stage. 

In recent years, researchers have extensively investigated technologies that use wearable devices to distinguish between patients with PD and healthy individuals [15,16], as well as between these patients and those with ET [17,18]. The use of novel technologies, such as machine learning, has substantially improved the accuracy of such diagnostic techniques. PD diagnosis is sometimes difficult, even for specialists, and is facilitated by the aforementioned techniques. In addition, researchers are investigating technologies to assess and monitor tremors using wearable sensors [19,20]. Monitoring reflects routine symptoms because it does not comprise a time-limited snapshot, unlike assessments at clinics and other facilities. In recent years, these evaluations have become possible and easier to handle with familiar devices, such as smartphones [21,22] and smartwatches [23,24].

**Figure 1:**
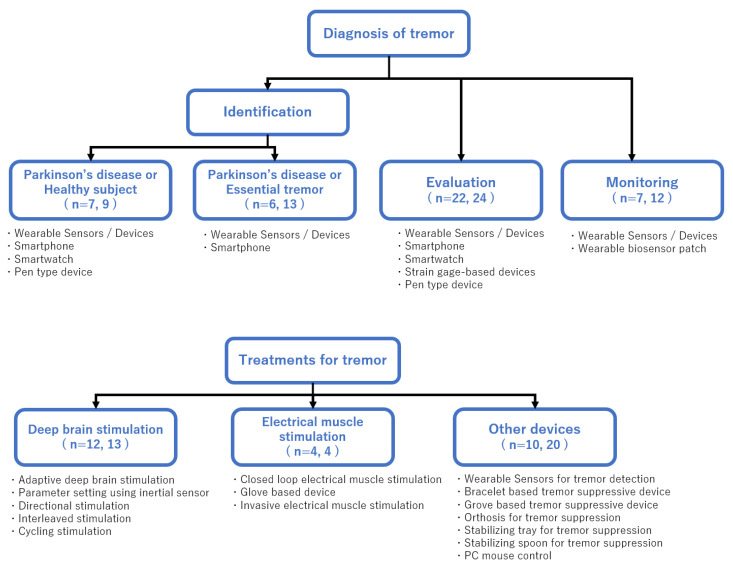
An overview of the diagnostic and treatment devices for tremor. The figure indicates the diagnosis and treatment of tremor, tremor type, and content. The numbers in parentheses show the breakdown of the number of search hits, with PubMed and Scopus listed in that order.

Because tremor is only one manifestation of PD (e.g., motor, and non-motor symptoms), the patient’s medical condition should be decided by evaluating all symptoms. Regarding tremors, pharmacotherapy is the primary treatment for PD. Approximately 30% of patients with tremors do not respond to pharmacological intervention or they experience intolerable secondary effects [25]. Deep brain stimulation (DBS) is another effective treatment for medically refractory tremors. An upcoming notable technique of DBS may be the detection of tremors using local field potential (LFP) [26,27]. This tremor detection technique will be available in adaptive DBS (aDBS), which operates on the principle of closed-loop interaction. Compared with DBS, electrical muscle stimulation (EMS) would be a safer treatment that can improve symptoms with fewer side effects. EMS also comprises a closed-loop system that detects tremors and stimulates them accordingly and is effective in suppressing tremors [28,29]. Another approach encompasses tremor suppression using orthosis [30,31]. A common method is to estimate the tremor and apply a reverse cancellation signal. Researchers are investigating techniques for tremor detection based on data acquired from inertial sensors and other sources [32,33], which is essential to avoid the risk of suppressing non-tremor movement. Treatment using mechanical devices in patients with PD has been reported to be effective and is widely accepted [34]. In this systematic narrative literature review, we will introduce recent technologies and research trends related to PD tremor diagnosis using devices, device-based tremor treatment, and orthosis for tremor control (Figure 1).

## 2. Materials and Methods

All searches were performed on PubMed and Scopus. The study screening was done independently by two reviewers, J.F. and R.M. We searched for “Parkinson disease” and “tremor” and “device”. English language literature in the past 10 years was reviewed. After removing duplicates, we included papers with at least one diagnostic outcome (identification, evaluation, or monitoring) or the treatments. Titles and abstracts were screened for eligibility by two researchers (J.F. and R.M.). The full texts were reviewed by the same researchers with any disagreement in study selection being resolved through discussion.

## 3. Results

The search yielded 268 results in PubMed and 253 in Scopus. There were 73 relevant articles in PubMed and 98 in Scopus. After removing the duplicate papers, two researchers (JF and RM) assessed the remaining articles. In total, 86 articles (distinguishing between PD and healthy individual, 7; distinguishing between PD and ET, 9; tremor evaluation, 23; tremor monitoring, 11; DBS, 15; EMS, 4; and other devices, 17) were compiled as a narrative literature review. There were only two randomized controlled trials in the selected literature: one article on DBS [35], and the other on EMS [28]. The number of literatures was too small to perform a robust systematic review for each category. Therefore, we conducted a systematic narrative review. Figure 1 shows the number of papers for each topic.

### 3.1. Tremor Diagnosis Using Devices

Previous studies have proposed measurable technologies and analysis methods for Parkinsonian tremor [22,36,37,38,39,40,41,42,43,44,45,46]. Researchers have used movement sensors, such as accelerometers and gyroscopes, and electromyography (EMG) for monitoring, qualifying, and detecting tremors, and differentiating between tremors due to PD, those due to other causes (such as ET), or those in healthy people. Furthermore, new smartphones and smartwatches with gyroscope and accelerometer functions can be used as accessible monitoring tools. This chapter presents research on the diagnosis and evaluation of tremors using these devices.

#### 3.1.1. Distinguishing between Patients with PD and Healthy Individuals, Using Devices

With regard to tremor diagnosis, distinguishing between ET and PD is sometimes challenging. Therefore, researchers are actively identifying ways to support differential diagnosis by device-based objective evaluation (Table 1). The first step in diagnosis is to distinguish a patient from a healthy individual. Giulia et al., used a wearable inertial sensor to discriminate between patients with PD and healthy participants [47]. Thirty-six patients with PD and 29 healthy controls performed the following seven motor tasks from the Movement Disorder Society-Sponsored Revision of the Unified Parkinson’s Disease Rating Scale (MDS-UPDRS) III wearing inertial sensors: resting tremor, postural tremor, rapid alternating hand movement, foot tapping, heel-to-toe tapping, timed up and go test (TUG), and a pull test. Of these endpoints, SVM was performed using highly relevant items, namely, tremor, bradycardia, pull test, and TUG, and was able to distinguish between patients with PD and healthy controls with a high accuracy of 97%. Channa et al., also developed the A-WEAR bracelet for diagnosis using 3D acceleration and gyroscopes, which accurately identified PD with a 91.7% probability by K-nearest neighbors [48]. Such research has been applied to smartphones and smartwatches. Kostikis et al., developed a smartphone-based tool to assess upper limb tremor in patients [22]. Using machine learning, the system correctly classified 82% and 90% of the patients and healthy participants, respectively, based on data from a smartphone’s accelerometer and gyroscope. Prototypes have also been developed using smartwatches [15]. This system was tested with artificial neural networks, random forests, and SVM, and trained on a sample comprising 192, 75, and 51 patients with PD, other movement disorders, and healthy participants, respectively. Artificial neural networks displayed the best results in distinguishing healthy participants from others, including those with PD and other movement disorders, with precision and recall of 0.94 (SD 0.03) and 0.92 (SD 0.04), respectively. Moreover, SVM demonstrated the best performance in distinguishing patients with PD from those with other motor disabilities, and healthy participants, with precision and recall of 0.81 (SD 0.01) and 0.89 (SD 0.04), respectively. Moreover, there are other validations of diagnostics using commercially available smartwatches [49]. A study which used the Apple Watch series 3 and 4, which are commonly distributed smartwatches, first evaluated their accuracy by comparing it with the Nanometrics seismometer. Both series 3 and 4 were confirmed to be accurate, with a maximum error of <0.01 Hz from the seismograph. The patient performed a test designed by a disability specialist to obtain acceleration data while wearing the smartwatch. Machine learning was used to discriminate between patients with PD, healthy participants, and those with motor impairments other than PD (ET, Parkinsonism, etc.). The machine learning classifiers used were as follows: SVM, CatBoost, multilayer perceptron, and simple deep learning architecture. SVM, CatBoost, and multilayer perceptron displayed a balanced accuracy of >80% and precision and recall rates of >90% for patients with PD and healthy participants. In an advanced task that distinguishes PD and non-PD motor impairment, the multilayer perceptron demonstrated a balanced accuracy, precision, and recall of 74.1%, 86.5%, and 90.5%, respectively. Thus, considerable identification accuracy can be achieved, even with consumer products. Another study used inertial data from a commercially available smartwatch to investigate eating behavior and evaluate the reduction in motor symptoms in PD [50]. They evaluated plate-to-mouth (PtM) in seven healthy participants and 21 patients with PD. PtM is a measure related to the average time for the hands to transfer food from the plate to the mouth when eating. Those with PD had higher PtM values than healthy participants. Furthermore, a model using PtM was used to classify patients and revealed precision, recall, and F1 (harmonic mean of fit and recall) of 0.882, 0.714, and 0.789, respectively. However, some of the aforementioned methods are difficult to use because they require expertise in system operation and maintenance. Junior et al., developed a device that can be combined with a regular pen as an approach for easier and simpler diagnosis [16]. It can distinguish patients with PD from healthy individuals through a simple diadochokinetic paper test, which assists in diagnosing the early stages of PD. The device was equipped with an accelerometer and gyroscope, and the acquired data were classified using linear discriminant analysis, logistic regression, classification and regression trees, K-nearest neighbors, SVM, and naive Bayes. The results confirmed that the overall accuracy was approximately 100% for multiple classifiers.

#### 3.1.2. Distinguishing between PD and ET Using Devices

The classic method of differentiating PD tremor from ET involves iodine-123-labelled N-omega-(flu-oropropyl)-2beta-carbomethoxy-3beta-(4-iodophenyl) tropane and iodine-123-labelled 2β-carbomethoxy-3β-(4-iodophenyl) tropane dopamine transporter imaging with single-photon emission computed tomography using nuclear imaging techniques [51,52,53]. However, its accuracy may be less than that of clinical diagnosis by movement disorder specialists [54]. In addition, nuclear imaging techniques are widely unavailable because they involve radiopharmaceuticals and are expensive and time-consuming. This warrants considering the mechanism of relatively inexpensive and widely available wearable devices to identify PD and ET. Despite an overlap between the frequency ranges exhibited by PD and ET tremors, the accelerometer power spectrum analysis signals can effectively distinguish between PD and ET [42,44,55,56,57,58]. Thanawattano et al., proposed a novel method for extracting temporal features based on variations in the frequency of tremors with state [42]. They attached six-axis inertial sensors to the index fingers of the participants and requested them to perform three tasks as follows: kinetic, postural, and resting. Each task took 10 s to complete. The elliptical regions of two-dimensional representations of the resting task for those with PD and ET were significantly different (*p* < 0.05). Locatelli et al., developed a small, low-cost, wearable device with an inertial sensor [59]. The device was worn on the wrist, and four standardized tasks were performed to acquire data and build a classification model, which achieved an average accuracy of >90%. Researchers have also used the acceleration from a smartwatch to identify PD and ET [44]. The use of the mean harmonic peak power obtained from the accelerometer could facilitate calculation of the optimal discrimination threshold by a receiver operating characteristic (ROC) analysis (sensitivity 90.9%, 95% CI 58.7–99.8%; specificity 100%, 95% CI 76.8–100%; and Cohen’s kappa = 0.91, SE = 0.08). In addition, the accuracy of the smartwatch was evaluated using an analog accelerometer and provided consistent estimates of the peak frequency and proportional harmonic power. Studies have also been conducted using smartphones: Woods et al., performed a task while holding a smartphone in the hand to obtain acceleration information [57]. This application used discrete wavelet transforms and SVM to classify the data and found an accuracy rate of over 96%. Barrantes et al., also used smartphones to identify PD and ET [17]. Patients with an undecided diagnosis were included in the evaluation and were re-evaluated after 1 year. For the experiment, smartphones were placed on the dorsal side of the hand, and recordings were obtained for epochs of 30 s at rest and 30 s during arm stretching. They calculated the ROC of the total spectral power to establish a threshold to separate participants with and without tremors. The results demonstrated an accurate diagnosis of PD or ET in 27 of 32 patients (84.38% discrimination accuracy). Of the patients with undecided diagnoses, all PD cases (two) and two of four ET cases were correctly classified. Duque et al., also performed machine learning classification using the linear acceleration of tremor recorded by the smartphone’s built-in accelerometer, and showed performances ranging from 90.0% to 100.0% sensitivity, and 80% to 100% specificity [60]. Thus, the smartphone, a familiar device, is expected to be utilized. Moon et al., evaluated the performance of several machine-learning methods, including neural networks, SVM, K-nearest neighbor methods, decision trees, random forests, and gradient boosting [18]. They used six inertial sensors (on the wrist, back of the foot, sternum, and hip) to analyze balance and gait characteristics to distinguish between PD and ET. The F1 score (harmonic mean of fit and recall), which is the most commonly used performance metric in machine learning, was 0.61, 0.59, 0.56, 0.55, 0.53, and 0.49 for neural networks, gradient boosting, random forest, SVM, decision tree, and K-nearest neighbors, respectively. It was superior to conventional logistic regression, thus confirming the usefulness of machine learning for diagnosis. Most studies were diagnostic, based on data obtained from inertial sensors, although some studies were conducted using EMG. A study investigating the EEG characteristics of resting tremor in patients with ET and PD confirmed that the parameter that best differentiated the two disorders was the pattern of muscle activation [61]. Vescio et al., developed a μEMG device worn on the wrist to record resting tremor [62]. Comparison with common EMG recordings confirmed a good correlation between tremor frequency (r = 0.93, *p* < 0.001) and phase difference (r = 0.92, *p* < 0.001). Thus, wearable devices have been used to classify PD and ET with high accuracy. Further validation may provide more efficient diagnostic and prognostic tools that can assist clinicians in decision-making processes.

### 3.2. Tremor Evaluation Using Devices

In addition to identification, it is important to quantitatively assess the degree of tremor. The UPDRS tremor scores are often used for tremor evaluation in clinical practice. Moreover, evaluation systems using wearable devices are often designed to correlate sensor measurements with the UPDRS. This section describes research on the evaluation of tremors using the aforementioned device.

#### 3.2.1. Tremor Evaluation Using Wearable Sensors

Raethjen et al., and Zhang et al., used EEG and EMG data to characterize tremors in patients with PD [63,64]. Currently, surface electromyography (sEMG) is the standard technique used for the characterization and monitoring of tremors in patients with PD [65]. Researchers have assessed the severity of tremors to determine the diagnostic usefulness of sEMG. They compared 30 patients with PD with a healthy age-matched control group by attaching bipolar sEMG to the biceps brachii muscle and evaluated muscle activity. The recurrence and determinism rates were significantly higher in the PD group than in the control group, and were correlated with the UPDRS scores [66]. Inertial sensors have been increasingly used in recent years [67,68,69,70]. A study comparing the accuracy of inertial sensors and EMG motion tracking showed that inertial sensors were more accurate [71]. Data-processing approaches vary across studies. Rigas et al., successfully estimated tremor severity based on acceleration acquired using accelerometers attached to body segments and features extracted from a hidden Markov model [72]. Using a gradient descent algorithm, Cai et al., isolated the acceleration caused by pure translational motion. A multiple regression model of UPDRS was created from the features extracted from these accelerations and angular velocities [73]. The performance of this model was r^2^ = 0.95 for resting tremor and r^2^ = 0.93 for postural tremor. Kim et al., developed SNUMAP, a wrist-mounted evaluation device with a three-axis accelerometer and gyroscope [74]. They trained recordings from 92 patients with PD using a convolutional neural network (CNN) to create an estimated UPDRS model. The results displayed an average accuracy of 85%, with a linearly weighted kappa of 0.85. CNNs could achieve higher accuracy than simple machine learning methods, such as SVM or regression. Such machine learning-based methods have been a trend in recent years, and numerous studies have been conducted. Wu et al., extracted characteristic values from acceleration signals in the time, frequency, and spectral domains, and tested multiple machine learning methods. The results showed that the neural network model was more accurate than the SVM, random forest, and multivariate linear regression models [75]. Another method using CNNs is to learn a convolved 2D image of the frequency response of the tremor signal [76]. This study used data acquired from an accelerometer-based wearable device attached to the patient’s upper extremity. The results showed an average accuracy of 91%, with a linearly weighted kappa of 0.91. Moreover, researchers have proposed an approach to estimate UPDRS using fuzzy inference rather than machine learning [77]. The fuzzy theory postulates that the truth value is not binary, true, or false but rather deals with all intermediate values. Moreover, it considers uncertainty. This method is scalable and easily tunable because it is modeled in a manner similar to the human inference process. Garza-Rodríguez et al., also used fuzzy inference to evaluate UPDRS from hand pronation/supination exercises, and found that in most cases the results were consistent with expert evaluation [78]. While wearable sensors have the advantage of monitoring patients in several situations, devices that do not require attachment to the patient are also useful while measuring behavioral tremors under specific conditions. This led to the development of Rehapiano, an ergonomically designed tremor evaluation device with strain gauges placed on a two-handed handle [79]. The performance evaluation also confirmed that the sensitivity was sufficient to quantify the tremor. The other product is the TREMITAS-System, a pen-type device with an accelerometer, 3D gyroscope, and 3D magnetometer [80]. This device was able to quantify tremors and was significantly correlated with UPDRS and the tremor research group essential tremor assessment scale subscores.

#### 3.2.2. Tremor Evaluation Using Smartphones and Smartwatches

Smartphones have become the most popular devices in recent years, and studies have been conducted on the evaluation of tremors using these devices. This is partly attributable to the rapid increase in their computing power. Lemoyne et al., used a common smartphone to evaluate tremor frequency in patients with PD [81]. Araujo et al., demonstrated good consistency between a clinically obtained EMG, and accelerometer data obtained using smartphone applications (Pearson > 0.8, *p* < 0.001) [82]. In this study, 22 patients with diagnoses of PD, ET, and Holmes’ tremor were tested, and three apps were evaluated. Bermeo et al., developed an Android application that could assess the status of patients with PD based on three tests in the MDS-UPDRS [83]. Kostikis et al., used a smartphone’s gyroscope and accelerometer to detect and quantify tremors. The smartphone detected data on hand tremors, and the UPDRS hand tremor scores revealed a good correlation (r > 0.7 and *p* < 0.01) [22]. These studies have often been conducted in controlled environments, such as laboratories; however, some studies were performed in free-living environments. Researchers have proposed an algorithm for tremor classification using a multiple-instance learning method based on smartphone acceleration to cope with noisy data, which demonstrated good classification performance [21]. van Brummelen et al., compared laboratory-grade and consumer product accelerometers and suggested that the amplitude at peak frequency varied among the sensors, indicating that distal worn sensors tended to measure higher amplitudes relative to proximal ones. Thus, the placement of sensors may be an important part of evaluating tremor amplitude [84]. Thus, tremors can be detected and evaluated with high precision without using dedicated sensors, besides having considerably lower hurdles for their use.

In addition to smartphones, several smartwatches have become popular in recent years, with the availability of numerous models. Smartwatches may be suitable for tremor assessment because they are worn on the wrists. Several studies evaluated tremors using sensors attached to the wrist. López-Blanco et al., quantified resting tremors by obtaining the parameters of tremor intensity from the root mean square of angular velocity acquired from a smartwatch [85]. Furthermore, they simultaneously performed a statistical analysis of the quantified data with the UPDRS-III score, which revealed a strong correlation with a Spearman’s correlation coefficient (ρ) of 0.81 (*p* < 0.001). In addition, satisfaction associated with the device was high. Tremors can also be classified using machine learning based on triaxial acceleration data from commercially available smartwatches [24]. Investigators have achieved high tremor detection performance using a multitasking CNN that uses both raw signals and spectral data representations as inputs. They are exploring data measurement characteristics necessary for the accurate detection of PD symptoms [23]. Following a comparative evaluation of commercially available smartwatches and measurement sensors with multiple functions, accelerometer data from the smartwatch alone were sufficient to detect tremors. A sampling rate ≥ 30 Hz was required to detect tremors using acceleration. In addition, they investigated the impact of the features used in machine learning (time, frequency, entropy, correlation, and derivative) on accuracy. Entropy was identified to be important for tremor detection. Entropy is computationally expensive and affects real-time performance and battery consumption. Taken together, tremor detection using smartwatches has reached a practical level and is expected to be utilized. The use of affordable wearable technology is less burdensome and the most useful approach for routine care and assessment of patients with PD.

### 3.3. Tremor Monitoring

Despite the variety of novel devices being designed to assess tremors at specific times, several studies have aimed at continuous monitoring. Assessments in clinics and other settings are time-limited and may not reflect routine symptoms. This warrants an evaluation with prolonged monitoring to accurately assess disease status. Such monitoring is also expected to be used as screening for the application of advanced treatments, such as DBS [86]. In the early studies, there was a type of study in which tests were taken several times a day [87]; however, in recent years, a continuous monitoring system has also been realized. Pulliam et al., attached motion sensors to the limbs and obtained data for six daily activities, such as eating and brushing teeth [88]. Assessments of 13 patients with PD revealed that the ratings by ROC curves were consistent with the clinician’s UPDRS-III ratings of the video recordings (ROC area > 0.8). Similarly, Hssayeni et al., measured tremor severity from activities of daily living with wrist- and ankle-mounted three-axis gyroscopic sensors; results from 24 patients with PD displayed the maximum correlation of 0.96 in gradient tree boosting [89]. Researchers have proposed wearable sensors attached to the wrist and chest combined with questionnaire-based assessment for continuous monitoring of PD symptoms in daily life [90]. Overlapping frequency components make it difficult to distinguish between daily activities and tremors; nonetheless, a method has been proposed for detecting tremors using a two-step algorithm [91]. Another device that can detect PD hand tremors from daily movements is the PD-Watch [92]. This device enables detection by checking for movement frequency and supination–pronation characteristics. The index calculated from 24 h of data obtained from this device was shown to correlate with the UPDRS score. Furthermore, a system was proposed as a machine learning approach to detect tremors in daily life data using a CNN and other techniques from a wearable accelerometer system worn on the wrist [19]. This technology enabled the quantification of the number of tremors in daily life. For a more user-friendly and complete sensor, researchers developed the biosensor patch NIMBLE (MC10, Inc., Lexington, MA, USA) with an accelerometer and myoelectric system [20]. It can adhere to the skin using adhesive stickers. In addition, the measurements can be wirelessly transmitted to a smartphone or tablet and a cloud server. Prediction scores using acquired data were within the range of ±1, with a probability of 91%. Moreover, their adhesion and safety were evaluated. Such techniques may allow for better treatment by assessing tremors at higher frequencies in daily activities.

### 3.4. Treatments for Tremor

#### 3.4.1. DBS

DBS has been established as the standard of care for patients with movement disorders, such as PD, ET, and dystonia. DBS is an effective and widely used treatment for these patients, and the majority of them achieve good clinical results following surgery [93,94,95]. DBS improves bradykinesia [93], gait freezing in PD [96,97], camptocormia [98,99], and tremor [100]. Moreover, it is a safer treatment with lower complication rates than stereotactic thalamotomy [101,102]. The subthalamic nucleus (STN) and internal globus pallidus (GPi) are the most common targets for PD stimulation [103]. A meta-analysis evaluating the effect of DBS on tremor suppression compared DBS ON and OFF conditions and found a significant standardized difference mean effect (effect size = 0.36; 95% CI = 0.316–0.395; *p* < 0.0001) [104]. The sum of UPDRS III, items 20 and 21, was used for this measure. These results indicate moderate effectiveness. Z-test results showed no significant difference in effect size between STN and GPi (*p* = 0.56). A 12-month follow-up study also confirmed its effectiveness in reducing action/postural tremor and resting tremor [105]. The results of this study also showed that the extent of tremor control by DBS for STN and GPi were equivalent; however, it may take longer to achieve the same tremor control effect when targeting GPi. It has also been found that the therapeutic effect varies with the stimulation frequency. Meta-analyses have shown that stimulation greater than 100 Hz STN has a greater effect on tremor. [106]. The method of electrode placement is also important. Diffusion tensor imaging and tractography guided lead placement have been shown to provide more stable placement and better tremor control compared to conventional methods of lead placement [35]. Other targets include the ventral intermediate nucleus of the thalamus (VIM), caudal zona incerta, and posterior subthalamic area, which have a striking effect in improving tremors [107,108,109,110,111,112]. Therefore, if tremor is the main problem, rather than bradykinesia for patients with PD, these targets are warranted to be considered as first choice. A study of 98 patients with PD and ET showed sustained improvement in tremor scores (UPDRS III, items 20 and 21; Fahn–Tolosa–Marin Tremor Rating Scale) with VIM stimulation (mean improvement, 70% and 66% at 1 year and 63% and 48% at >10 years, respectively, *p* < 0.05) [112]. There was no significant loss of a stimulation effect over time (*p* > 0.05). Thus, the effects of DBS are long-lasting. Tremor can be controlled by maintaining the activities of daily living, and there was high patient satisfaction during the 10-year follow-up [113]. However, DBS is not effective in all patients, and patients need to determine whether they are appropriate candidates.

##### aDBS

Advances in DBS technology are ongoing, and novel research and development are underway. aDBS is one of the most innovative techniques. An aDBS device operates on the principle of closed-loop interaction, which can determine the effect of stimulation and adjust it, in response to the observed effect. LFPs are used as biomarkers to achieve a closed loop in aDBS. aDBS with LFPs has the advantage of being achieved by the online analysis of deep brain recordings, without the need for additional measurement channels. It is effective and is currently being used to treat patients with PD [114,115,116,117].

With regard to tremor suppression, an aDBS device has not been used clinically. Performing DBS only after the appearance of symptoms may reduce battery consumption. Power consumption is important because battery replacement requires surgery. aDBS necessitates the detection of tremors from the LFP. Reliable symptom detection is important for the implementation of aDBS. Tremor-related activity occurs throughout the motor network [118,119]. Specifically, it includes the basal ganglia, thalamus, cerebellum, and primary motor cortex, which coherently respond at tremor frequencies of 3–7 Hz upon their presentation [120]. Other findings include an increase in low gamma power (31–45 Hz) [121,122] and changes in high-frequency fluctuations in the subthalamic nucleus [123]. Advanced techniques, such as machine learning, are required to capture the aforementioned complex features. Hirschmann et al., used a hidden Markov model to classify tremors [27]. They obtained the LFP from the STN of 10 patients with PD, which was evaluated using four frequency domains (power at the individual tremor frequency ± 1 Hz, beta power, low gamma power, and high-frequency oscillations power ratio). The results indicated a mean area under the ROC curve of 0.82 and a SD of 0.15. Furthermore, it displayed good accuracy, even without training, for individual patients. High-frequency domain is the most useful feature for detecting tremors. Camara et al., developed a system that simultaneously recorded LFP in the subthalamic nucleus and electromyographic activity in the forearm and used fuzzy inference to detect tremors based on the relationship between the signals [124]. The system displayed 100% accuracy in detecting tremors in four of 10 patients with PD and attained >98.7% accuracy in seven patients. A complete system can be built within the DBS hardware by extending this work to synchronization between the thalamic LFP and other brain signals. Shah et al., proposed a tremor detection system using a logistic regression-based classifier [26]. The system was trained by extracting various features from the frequency and time domains of LFPs. The classification performance ranged from an AUC of 0.67 to 0.93, thereby indicating that the power between 31 Hz and 45 Hz was the most discriminating feature. A multi-feature neural-network-based tremor detector was proposed as a machine-learning-based approach [125]. It achieved an accuracy > 86% in four of eight patients. These technologies will help an aDBS system realize accurate treatment.

##### The Optimization of Stimulation Parameters

The optimization of stimulation parameters is important for the efficient use of DBS. Setting general DBS parameters often relies on subjective evaluation, which may not yield optimal effects. In addition, multiple parameters, such as frequency, pulse width, and amplitude, must be set appropriately within the time constraints. Thus, researchers proposed next-generation methods to quantify tremors using wearable sensors as objective indicators for determining stimulation parameters. Pulliam et al., proposed an algorithm in which motion sensors, including accelerometers and gyroscopes, were attached to the fingers to acquire motion data, which were subsequently used to set the DBS parameters [126]. There were two algorithms, as follows: one to maximize the treatment effect and the other to optimize the battery life. The algorithm that maximized the treatment effect reduced motor symptoms by 13%; however, it increased the stimulus amplitude, compared with the usual setting method. In contrast, the algorithm that optimized battery life successfully reduced the stimulus amplitude by an average of 50% while maintaining the level of therapeutic effect. Currently, the intraoperative parameter setting is subjectively performed. A system was developed to assist in electrode placement and test stimulus settings during DBS implantation surgery for awake patients. The system facilitated the quantitative real-time visualization of neural activity recorded by microelectrode and motor symptoms, such as tremors, recorded by an inertial measurement unit during surgery [127]. Dai et al., also developed a glove-type system that uses an inertial measurement unit and force sensitive resistor to measure the immediate effects of DBS by tremors, bradykinesia, and rigidity assessments [128]. In addition, highly functional inertial sensors with conformal, wireless, and data upload functions, and the Food and Drug Administration (FDA)-approved BioStamp nPoint, have been developed [129]. These technologies will realize next-generation methods of optimizing stimulation parameters in clinical settings.

#### 3.4.2. EMS

Invasive devices, such as DBS electrodes, are a good option; however, the risk of adverse events associated with the procedure should be carefully assessed. It is important to identify novel and safe treatment options with fewer side effects, thus emphasizing the need for non-invasive devices. EMS may be one such method. However, it has not received FDA, CE Mark, or any other accreditation.

##### EMS Controlled by Motion Detectors

EMS, used to alleviate resting tremor, is based on modifications by changes in peripheral mechanical conditions, external joint motion, or EMG [130]. Jitkritsadakul et al., developed a glove-shaped portable device that detected and suppressed tremors [28]. It consisted of three components, as follows: a glove with an embedded inertial sensor and an EMS module, a control box that can be worn on the waist belt, and an Android smartphone. An inertial sensor attached to the glove was used to detect and stimulate tremors. EMS was performed via two electrodes placed over the short thumb abductor muscle and the first and second dorsal skeletal muscles. They evaluated the performance of this device using a double-blind, 1:1 pair-designed, randomized, placebo-controlled design in 30 patients with PD. The tremor glove effectively suppressed intractable resting hand tremors in these patients, without serious adverse events. Specifically, they identified a significant reduction in the root mean squared angular velocity (as a percentage) in every axis, in peak magnitude in the axis (x-, y-), and in UPDRS tremor scores (glove: 5.27 ± 2.19, sham: 4.93 ± 2.37) during stimulation with Tremor’s glove, compared with the sham groups (*p* < 0.05, each). Gallego et al., developed a device that integrated neurostimulation electrodes, gyroscopes, and control electronics [131]. It analyzed the characteristics of the tremor (instantaneous amplitude and frequency) from the gyroscope recordings and regulated the level of muscle co-contraction by injecting current into the antagonist pair, as appropriate. They obtained significant attenuation of the tremor (*p* < 0.001) in patients with PD and ET, reducing its amplitude to 52.33 ± 25.48%.

##### EMS Controlled by EMG Signals

Researchers have proposed a method for detecting tremors from the EMG signals of muscles. Dosen et al., proposed a method of detecting tremors from the EMG of the muscles causing the tremor and counteracting it by applying an out-of-phase electrical stimulation to a similar muscle [132]. The device was evaluated in four and two patients with PD and ET, respectively, and demonstrated an average tremor reduction of 46% to 81% and 35% to 48% in the five patients, respectively. In one patient, the system did not attenuate the tremor. Myoelectric sensors implanted in muscles have been developed to improve diagnostic accuracy [133]. The sensor can acquire EMG signals near muscle fibers, and the implantable system ensures a stable relationship between the source and electrodes. It has the advantage of being unaffected by external factors, such as sweat. Intramuscular electrodes can be placed using a hypodermic needle. These electrodes usually have only the function of a single recording; however, in recent years, investigators have developed multichannel electrodes made of thin polyimide films [134,135,136]. In addition, a device that not only records, but also simultaneously stimulates, has been developed [29]. It was built on a polyimide substrate and comprises 12 recording sites and three stimulation sites made of platinum. This device was tested on six patients with ET and three healthy participants to assess basic information, such as perceptual thresholds and current limits. Furthermore, the application of this electrode to the system created by Dosen et al., [132] suppressed tremors and wrist angles by an average of 58%.

#### 3.4.3. Other Devices

Common methods of tremor suppression include estimating the tremor and applying an opposite cancellation signal; however, mechanical suppression poses the risk of suppressing movements that are not tremors. This necessitates developing a technique to accurately detect tremors. Machine learning has been used in recent years to achieve a significantly higher tremor prediction performance [32,137]. Ibrahim et al., proposed a method for detecting tremors and voluntary motion based on neural networks [32]. They trained the network using the acceleration and angular velocities obtained from patients with PD using an inertial measurement device. Problems associated with such tremor detection in a wearable tremor suppression device include the inability to adapt to the nonlinear behavior of the tremor and the delay in tremor prediction, which reduces the performance of suppression. To solve these problems, a modified version was proposed that can learn the correlation and nonlinearity between tremors and voluntary movements and make predictions with minimal delay [33]. They created a task- and user-independent generalized model, which achieved an average estimation accuracy of 99.2%. The average future spontaneous motion prediction percentage accuracies at 10, 20, 50, and 100 steps ahead were 97.0%, 94.0%, 91.6%, and 89.9%, respectively. Moreover, the prediction time at 100 steps ahead was 1.5 ms. Delay reduction was achieved while maintaining an accuracy similar to that in previous studies. Additionally, TremorSense was developed to classify tremor types [138]. TremorSense consists of a wrist-mounted accelerometer and gyroscope that uses an 8-Layer CNN model to classify PD rest, posture, and action tremors with 94% accuracy. These algorithms would be useful for orthoses and tools for suppressing tremors. The devices introduced here have not received FDA or CE marking.

##### Tremor Suppression Using Orthosis

Several studies have used suppressive orthoses for tremor suppression [139,140] Herrnstadt and Menon developed a one-degree-of-freedom elbow brace that can be worn by people with tremors [141]. This system consisted of a suppression motor, gears, sensors, including force transducers and encoders, and braces on the upper arm and forearm. They evaluated the brace in nine patients diagnosed with mild to severe tremors, including PD, and observed a 94.4% (*p* < 0.001) reduction in the mean power of the tremor [142]. This type of tremor-suppression device requires a power supply and is termed an active device. In contrast, researchers have developed passive devices that operate by damping or absorbing vibration energy [143]. Buki et al., developed a passive device based on energy absorption, termed a Vib bracelet [30]. This device absorbs vibrations in the frequency range associated with tremors using the principle of a dynamic vibration absorber. This technology is widely used to absorb vibrations caused by earthquakes in bridges and high-rise buildings. It has a simple structure, weighs 280 g, and has a small and lightweight outer radius of 57 mm. The evaluation of the mechanical forearm enabled attenuation of the vibration in the range of 4 Hz to 5.75 Hz, with an amplitude attenuation of 86% (approximately one in 7.3) at 4.75 Hz. Further performance improvement can be achieved by personalizing the device according to the frequency of the tremor. Faizan et al., developed a passive bracelet-type device [31] that comprised a dual-parallel configuration passive vibration absorber. Their theoretical evaluation revealed that the device reduced the amplitude of angular motion of the wrist by 57.25%. Furthermore, an evaluation of patients with PD confirmed that rectangular sketching partially improved the tremors while using the device. While most of these studies have targeted wrist tremor, a glove-type device that independently controls tremor in each finger joint has also been proposed [144,145]. This device is designed to manage tremor in the index metacarpophalangeal joint, thumb metacarpophalangeal joint, and the wrist. Results show overall suppression of 73.1%, 80.7%, and 85.5% in resting tremor, 70.2%, 79.5%, and 81% in postural tremor, and 60.0%, 58.7%, and 65.0% in kinetic tremor in the index finger metacarpophalangeal joint, the thumb metacarpophalangeal joint, and the wrist, respectively. In addition, Wanasinghe et al., developed a lighter and less bulky glove-type device based on layer jamming [146]. When a vacuum is supplied to the layer jamming elements, which contain a stack of attached layers, this device increases the stiffness of the glove and suppresses hand tremor. An assessment of 11 tremor patients revealed mean frequency power reductions of 41.74, 41.99, and 24.7% for the index and middle fingers and in grasping, respectively, with a maximum power reduction of 59.15%. The above-mentioned studies are examples of reports on the impacts of engineering solutions.

##### Tools with Tremor Control Function

Additional approaches include research that incorporates a mechanism to suppress unintended movements in tools rather than the tremor itself. For example, researchers developed a tray to transport objects, which included a vibration stabilization function [147]. This tray includes a mechanical platform and an electronic system to suppress the vibration of the base plate. It is stabilized by controlling three servomotors in a direction that counteracts the changes based on the data acquired by the inertial sensors. Some tableware contains a tremor control function. The Liftware Steady^TM^ (Liftware, Inc., San Francisco, CA, USA) comprises an electronically controlled stabilizing handle and numerous attachments, including a spoon, fork, and spork, to facilitate eating for patients with tremors. A pilot study demonstrated an improvement in tremor with the Liftwear Steady^TM^ using the Fahn–Tolosa–Marin Tremor Rating Scale [148]. In addition, investigators have attempted to use such spoons for tremor assessment [149]. In this study, the tremors were assessed using a linear model trained from motion signals that recorded the tremors. A modified Fahn–Tolosa–Marin scale was used for the assessment, and the correlation coefficient between the expert rating and the model score was 0.91 (*p* < 0.001). It demonstrated practical accuracy and can be used for daily objective monitoring. In addition, technologies have been proposed to assist in computer mouse control [150]. This method uses adaptive path smoothing via the B-spline to provide a smooth mouse path.

**Table 1:** Diagnosis of Parkinson’s disease tremor using wearable devices.

	Reference	Method	Performance
Distinguishing between Patients with PD and HC	Channa et al., 2021 [48]	Developed A-WEAR bracelet including 3D acceleration and gyroscope. During the clinical evaluation, based on UPDRS, upper limb motor activities employed by the neurologist were performed and temporal and spectral features were acquired by A-WERE. Machine learning was performed on this data to discriminate between healthy controls and PD patients.	Accurately identified 91.7% by K-nearest neighbors.
	Varghese et al., 2021 [49]	The patients performed a test designed by a disability specialist while wearing a smartwatch to obtain the acceleration data. Machine learning was used to discriminate between patients with PD, healthy participants, and those with motor impairments other than PD (e.g., ET, Parkinsonism).	SVM, CatBoost, and multilayer perceptron revealed a balanced accuracy > 80% and precision and recall rates > 90% for those with PD and healthy participants. In a more advanced task, the multilayer perceptron displayed a balanced accuracy, precision, and recall of 74.1%, 86.5%, and 90.5%, respectively, while distinguishing PD from non-PD motor impairment.
	Vescio et al., 2021 [62]	A μEMG device was developed to be attached to the wrist. Resting tremor was recorded using the μEMG device and a common EMG.	Comparison of the two EMGs revealed good correlation between tremor frequency (r = 0.93, *p* < 0.001) and phase difference (r = 0.92, *p* < 0.001).
	Di Lazzaro et al., 2020 [47]	65 patients (36 patients with PD and 29 HC) were fitted with inertial sensors and performed seven MDS-UPDRS III motor tasks as follows: rest tremor, postural tremor, rapid alternating hand movement, foot tapping, heel-to-toe tapping, TUG, and pull test. Relief ranking and Kruskal–Wallis feature-selection were used to extract the relevant tasks, and SVM was used to identify them.	SVM was performed using tremor, bradykinesia, pull test, and TUG. It could distinguish PD from HC with a high accuracy of 97%.
	Junior et al., 2020 [16]	Performed diadochokinesis test using a pen with an attached sensor device. The device was equipped with an accelerometer and gyroscope, and the acquired data were classified using Linear Discriminant Analysis, Logistic Regression, Classification and regression trees, K-Nearest Neighbors, SVM, and Naive Bayes.	Overall accuracy was approximately 100% for multiple classifiers.
	Kyritsis et al., 2020 [50]	Used a smartwatch to calculate the PtM, a measure related to the average time spent in transferring food from the plate to the mouth during eating. The classification was performed using a collected dataset of 28 participants (seven healthy controls and 21 patients with PD).	PtM generated precision, recall, and F1 of 0.882, 0.714, and 0.789, respectively, towards classifying the eating of meals from the patients with PD and healthy controls.
	Locatelli et al., 2020 [59]	An inertial sensor was attached to a wearable device on the wrist and data was acquired by performing four tasks to standardize the device. Applied supervised learning methods to build several classification models.	The classifier built from inertial-based, power-related features performed best, achieving over 90% accuracy.
	Moon et al., 2020 [18]	524 patients with PD and 43 patients with ET were fitted with six inertial sensors (wrist, back of the foot, sternum, and hip) and distinguished based on balance and gait characteristics collected from these sensors.	The neural network generated the highest F1-score (0.61), followed by gradient boosting (0.59), random forest (0.56), SVM (0.55), decision tree (0.53), and K-nearest neighbor (0.49).
	Varghese et al., 2020 [15]	Developed a smartwatch-based prototype system. This system was tested with artificial neural networks, random forests, and SVM, and was trained on a sample of 192, 75, and 51 patients with PD, other movement disorders, and healthy participants, respectively.	The artificial neural networks demonstrated the best results for distinguishing healthy individuals from those with PD and other movement disorders, with a precision and recall of 0.94 (SD 0.03) and 0.92 (SD 0.04), respectively. The SVM displayed the best results while distinguishing patients with PD from those with other motor disabilities and healthy individuals, with a precision and recall of 0.81 (SD 0.01) and 0.89 (SD 0.04), respectively.
	Kostikis et al., 2015 [22]	Quantified tremor by calculating a series of metrics using signals from a smartphone’s accelerometer and gyroscope in a small-scale clinical study comprising 25 patients with PD and 20 age-matched healthy participants.	Accurately classified 82% and 90% of patients with PD and healthy participants, respectively.
Distinguishing between Patients with PD and ET	Loaiza Duque et al., 2019 [60]	Dynamic features were extracted from the linear acceleration of tremors recorded by the smartphone’s built-in acceleration sensor. Classification was performed by machine learning.	Sensitivity of 90.0% to 100.0% and specificity of 80% to 100% were achieved.
	Barrantes et al., 2017 [17]	Measured tremor by attaching a smartphone to the dorsum of the hand. It calculated the ROC of the total spectral power to establish a threshold to separate participants with and without tremor.	The smartphone generated an accurate diagnosis of PD or ET in 27 of 32 patients (84.38% discrimination accuracy). Of those with an undecided diagnosis, all patients with PD (two) and two of four patients with ET were correctly classified; one patient with PD plus ET was classified as PD.
	Thanawattano et al., 2015 [42]	Attached six-axis inertial sensors to the index fingers of participants and requested them to perform three tasks, as follows: kinetic, postural, and resting tasks.	The elliptical regions of two-dimensional representations of the resting task for those with PD and ET were statistically significantly different (*p* < 0.05).
	Bhidayasiri et al., 2014 [58]	Developed a low-cost device consisting of a 3-axis accelerometer and a 3-axis gyroscope. Performed three tremor tasks including resting, postural, and motor tremor according to the motor section of the UPDRS.	Patients with ET showed significantly higher peak frequency during y-axis postural movements than patients with PD (*p* < 0.05).
	Wile et al., 2014 [44]	Obtained the mean harmonic peak power from the accelerometer of the smartwatch.	Using the mean harmonic peak power obtained from the accelerometer, the optimal discrimination threshold could be calculated by ROC analysis (sensitivity 90.9%, 95% CI 58.7–99.8%; specificity 100%, 95% CI 76.8–100%; and Cohen’s kappa = 0.91, SE = 0.08)
	Woods et al., 2014 [57]	Six tasks were performed with the smartphone in the hand, eyes open, eyes closed, etc. Each task was performed for 10 s and was performed with each of the two hands.	Used discrete wavelet transforms and SVMs to classify the data and found an accuracy rate of 96.4%.

PD: Parkinson’s disease; HC: healthy controls; MDS-UPDRS: Movement Disorder Society-Sponsored Revision of the Unified Parkinson’s Disease Rating Scale; TUG: Timed-Up-and-Go test; SVM: Support Vector machines; ET: essential tremor; PtM: plate-to-mouth; ROC: receiver operating characteristic.

## 4. Discussion

In this systematic narrative literature review, we introduced recent technologies and research trends related to PD diagnosis and treatment related to tremors. Initially, we reviewed studies that distinguished between patients with PD and healthy individuals and between PD and ET. Objective evaluation with respect to diagnosis is difficult, and conventional methods using nuclear imaging technology and other methods may not be as accurate as those used by movement disorder specialists [54]. Hence, misdiagnosis owing to confusion between PD and ET may occur in 20% to 30% of cases [13,14]. Therefore, device-based diagnostic technology that is both simple and highly accurate is important. Device-based diagnosis is becoming more realistic as technology advances and sensors become smaller and less expensive. Moreover, machine learning has been used in recent years to achieve a high degree of accuracy [18]. In addition to distinguishing between healthy individuals and patients with PD, it enables distinguishing the tremors of PD and ET [17,18]. While such diagnostic devices are expected to be utilized in the medical field, the early detection of PD is expected to be realized in the future through screening at the population level by using familiar devices, such as smartphones and smartwatches, to differentiate patients with PD from healthy individuals. As shown in Table 1, a device-dependent diagnosis may result in an objective diagnosis with a high degree of accuracy, close to 100% in some cases. Clinicians who are not usually engaged in tremor diagnosis could find these devices especially useful to enhance their diagnostic accuracy. However, they are not perfect and should be used as an adjunct to clinical evaluation at this time. In addition to diagnosis, it is important to assess the degree of tremors quantitatively. The UPDRS is often used for tremor evaluation in clinical practice; however, this score depends on a rater’s subjective assessment. An evaluation system using a wearable device might enable an accurate and objective evaluation of the degree of tremor [74,77]. Many of the studies presented aim to replicate expert UPDRS evaluations with devices [73,76,78]. For example, sensor information for expert evaluations can be modeled using machine learning. While this is quite useful in current practice, future research would include an absolute evaluation that is not limited to the UPDRS. This would allow for more detailed evaluation. Moreover, these devices are currently limited to lab-based research and are not yet in a state where clinicians can use them. Practical use in a clinical setting will evoke new issues. In addition, researchers have investigated approaches to evaluate disease states by monitoring daily routines using wearable sensors [19,20]. Monitoring techniques have also not yet reached general practical use. This requires the implementation of a simpler, more sophisticated system, as it may be used by patients who are not familiar with electronic devices. Data storage in the cloud, security, and other data handling issues also need to be addressed. Inexpensive, easy-to-use, high-performance devices, such as smartphones and smartwatches, are likely to be key to the progress and diffusion of monitoring technology. Researchers have also explored the potential of smartphones [21,22] and smartwatches [23,24] to decrease hurdles in evaluating medical conditions in the future. This has been verified using actual products and is close to practical use. Thus, research is now beginning to be conducted at the practical level, in addition to lab-level research. Some studies comparing the performance of smartwatches with research-level sensors have shown that they are sufficient for tremor detection [24], and the technical problems that have been overcome in recent years should accelerate practical application. Furthermore, it is also important to distinguish between dyskinesia and tremor in the advanced stage of PD. Advances in this technology are expected in the future.

Many studies have focused on DBS [26,27], EMS [28,29], and orthoses for tremor suppression [30,31]. There has been remarkable progress in alleviating tremors using DBS. In recent years, advanced information technologies, such as machine learning, have substantially improved [26,27]. This could further increase the potential of DBS in managing movement disorders. In addition, DBS has been established as the standard of care and is one of the most reliable treatments, with measures taken to ensure long-term safety [113]. Furthermore, new technologies such as aDBS and stimulation methods have been actively researched, developed, and put into practical use, and future developments are expected. However, DBS is an invasive process; hence there is a need for the development of non-invasive techniques that have a lower risk of adverse effects. One such technique, closed-loop EMS, stimulates muscles when it detects tremors from EMG. This feedback approach has been reported to significantly suppress tremors [29,132]. Interestingly, invasive EMS that requires surgical intervention has also been developed [29] However, this should be used in patients only after a thorough risk–benefit analysis. There has been substantial research and development in the use of orthoses and other devices to mechanically suppress tremors. In this treatment, it is important to detect tremors with a high degree of accuracy for adequate suppression, because there is a risk of suppressing movements that are not tremors. This can be accomplished by using machine learning [32,33]. The use of orthosis and tools is shown to suppress tremors under certain conditions, but not drastically. In addition, although most studies have focused on wrist tremor, there is a need to address various types of tremors in other parts of the body. These devices need to be evaluated in daily use regarding comfort, usability, and habituation. The effects of these treatment devices on tremor are summarized in Table 2. Thus, the tremor suppression effect of DBS is significant, and long-term evaluations of more than 10 years have been conducted. Some studies have confirmed that EMS is effective in suppressing tremor by 50% or more, so a certain level of effectiveness can be expected. Orthosis has been devised in a variety of ways and may have an even greater inhibitory effect. While some orthotic restraint devices and tools, such as Liftware Steady^TM^, have been commercialized, most orthotics are at the stage of lab-based research. There is a need to develop sophisticated products that are easy for patients to use. For example, there are concerns about difficulty in wearing and operating devices because of tremors. It is important to design products that take these concerns into account. In addition, an interface that is easy and intuitive to use is necessary. It is also important for patients to be aware of these products.

In this way, although various studies on devices and their effects have been reported, few studies have been conducted to date with a high level of evidence; therefore, large-scale studies and randomized controlled trials are needed. Except for DBS and Liftware Steady^TM^, no FDA certification or CE mark has been acquired for these devices against tremors, which is another hurdle for clinical use. Furthermore, the validation of performance and improvement in tremor are not standardized, making it difficult to compare studies. Another issue is the phenomenon of habituation. These devices are effective in the short term, and investigators have conducted long-term assessments as well. In addition, most of the studies have focused on hand tremors. It is important to develop techniques that alleviate axial tremors, such as head and vocal tremors.

Limitations of this literature review include simple search terms. Many pertinent papers were extracted; however, there may be missing, relevant articles. Due to the small number of randomized controlled trials, it was impossible to conduct an objective and rigorous evaluation through a systematic review.

## 5. Conclusions

This review summarizes the diagnosis and treatment of tremor in PD using devices. Research trends and issues in devices for tremor have been identified. Many studies have demonstrated that the diagnosis and evaluation of tremor in patients with PD can be highly accurate using machine learning algorithms. Wearable devices can be prognostic tools that assist clinicians in decision-making processes. The use of wearable mobile devices enables the monitoring of routine symptoms. Tremors can be detected and evaluated with high precision without using dedicated sensors, with considerably lower hurdles for their use. DBS has been established as the standard of care for patients with PD. In recent years, aDBS and optimization of stimulation parameters have been studied to further improve treatment efficacy. EMS, orthosis, and tools for tremor suppression are still in the experimental stage. Other than DBS, no devices are in practical use for tremor treatments. To acquire high-level evidence, large-scale studies and randomized controlled trials are needed for these devices.

## Figures and Tables

**Table 2:** Effects of therapeutic devices on tremor suppression.

Device	Invasive/Non-Invasive	Reference	CE Marking and FDA Certification	Efficacy
Deep brain stimulation directly targeting the dentatorubrothalamic tract with tractography	Invasive	Low et al., 2019 [35]	CE marking and FDA approved	60 months post- surgery, the Fahn–Tolosa–Martin tremor rating, treated tremor score for arm and leg, and treated tremor score for the arm were 80.72%, 93.89% and 93.35% better than conventional lead insertion.
Deep brain stimulation of the subthalamic nucleus and internal globus pallidus	Invasive	Wong et al., 2019 [104]	CE marking and FDA approved	A meta-analysis using the sum of the UPDRS III, items 20 and 21, found effect size = 0.36; 95% CI = 0.316–0.395; *p* < 0.0001.
Deep brain stimulation of the ventral intermediate nucleus of the thalamus	Invasive	Cury et al., 2017 [112]	CE marking and FDA approved	Sustained improvement in tremor score (UPDRS III, items 20 and 21; Fahn–Tolosa–Marin tremor rating scale). Mean improvement, 70% at 1 year and 63% at >10 years, *p* < 0.05.
Electrical muscle stimulation	Invasive	Muceli et al., 2019 [134]	Not approved	Suppressed tremors and wrist angles by an average of 58%.
	Non-invasive	Jitkritsadakul et al., 2017 [28]	Not approved	Significant reduction in the root mean squared angular velocity (as a percentage) in every axis, peak magnitude in the axis (x-, y-), and UPDRS tremor scores (glove: 5.27 ± 2.19, sham: 4.93 ± 2.37).
		Dosen et al., 2015 [132]	Not approved	Evaluated in four and two patients with PD and ET, respectively, and demonstrated an average reduction in tremor of 46% to 81% and 35% to 48% in the five patients.
		Gallego et al., 2013 [131]	Not approved	Significant attenuation of tremor (*p* < 0.001) reducing its amplitude to 52.33 ± 25.48%.
Orthosis	Non-invasive	Wanasinghe et al., 2021 [146]	Not approved	Revealed a mean frequency power reduction of 41.74, 41.99, and 24.7% for the index and middle fingers and in grasping, respectively, with a maximum power reduction of 59.15%.
		Zhou et al., 2021 [144]	Not approved	Overall suppression of 73.1%, 80.7%, and 85.5% in resting tremor, 70.2%, 79.5%, and 81% in postural tremor, and 60.0%, 58.7%, and 65.0% in kinetic tremor in the index finger metacarpophalangeal joint, the thumb metacarpophalangeal joint, and the wrist, respectively.
		Faizan et al., 2020 [31]	Not approved	Reduced the amplitude of angular motion of the wrist by 57.25%.
		Buki et al., 2018 [30]	Not approved	Enabled attenuation of the vibration in the range of 4 Hz to 5.75 Hz, with an amplitude attenuation of 86% (approximately one in 7.3) at 4.75 Hz.
		Herrnstadt and Menon 2016 [141]	Not approved	Braces on the upper arm and forearm. Observed a 94.4% (*p* < 0.001) reduction in the mean power of the tremor.

PD: Parkinson’s disease; UPDRS: Unified Parkinson’s Disease Rating Scale; ET: essential tremor; FDA: Food and Drug Administration.

## Data Availability

Not applicable.
